# Secreted and surface proteome and transcriptome of *Opisthorchis felineus*

**DOI:** 10.3389/fpara.2023.1195457

**Published:** 2023-10-10

**Authors:** Yide Wong, Mark S. Pearson, Olga Fedorova, Vladimir Ivanov, Ekaterina Khmelevskaya, Bemnet Tedla, Buddhika Jayakody Arachchige, Sarah Reed, Matt Field, Thewarach Laha, Alex Loukas, Javier Sotillo

**Affiliations:** ^1^ Australian Institute of Tropical Health and Medicine, James Cook University, Cairns, QLD, Australia; ^2^ Centre for Tropical Bioinformatics and Molecular Biology, James Cook University, Cairns, QLD, Australia; ^3^ Department of Faculty Pediatrics, Federal State Budget Educational Institution of Higher Education, Siberian State Medical University, Ministry of Healthcare of Russian Federation, Tomsk, Russia; ^4^ Laboratory of Biological Models, Federal State Budget Educational Institution of Higher Education, Siberian State Medical University, Ministry of Healthcare of Russian Federation, Tomsk, Russia; ^5^ Central Research Laboratory, Federal State Budget Educational Institution of Higher Education, Siberian State Medical University, Ministry of Healthcare of Russian Federation, Tomsk, Tomsk, Russia; ^6^ Mass Spectrometry Facility, Centre for Clinical Research, University of Queensland, Brisbane, QLD, Australia; ^7^ College of Public Health, Medical and Veterinary Science, James Cook University, Cairns, QLD, Australia; ^8^ Immunogenomics Lab, Garvan Institute of Medical Research, Darlinghurst, NSW, Australia; ^9^ Menzies School of Health Research, Charles Darwin University, Darwin, NT, Thailand; ^10^ Department of Parasitology, Faculty of Medicine, Khon Kaen Universit, Khon Kaen, Thailand; ^11^ Parasitology Reference and Research Laboratory, Centro Nacional de Microbiología, Instituto de Salud Carlos III, Majadahonda, Madrid, Spain

**Keywords:** *Opisthorchis felineus*, proteomics, secretome, tegument, microvesicles (MVs), liver fluke

## Abstract

**Introduction:**

*Opisthorchis felineus*, *Opisthorchis viverrini*, and *Clonorchis sinensis* are the most medically important species of fish-borne zoonotic trematodes. *O. felineus* is endemic to the river plains of Western Siberia and Eastern Europe, and it is estimated that more than 1.6 million people could be infected with this parasite. Chronic opisthorchiasis may lead to significant gastrointestinal and hepatobiliary pathology. This study aimed to identify and characterize proteins from the secreted and tegumental proteomes of *O. felineus*.

**Methods:**

Adult flukes were collected from experimentally infected hamsters and cultured in vitro in serum-free media. We extracted proteins from different compartments of the *O. felineus* secretome, including (i) soluble excretory/secretory (ES) products; (ii) secreted 15K-extracellular vesicles (EVs); and (iii) tegument.

**Results:**

We also generated a transcriptome using long-read sequencing, and when this was combined with high-resolution mass spectrometry, sodium dodecyl sulfate–polyacrylamide gel electrophoresis (SDS-PAGE) separation, and protein digestion, we identified 686, 894, 389, 324, and 165 proteins from the ES, 15K-EV, and the three sequentially extracted tegument (TEG) protein fractions, respectively. We conducted in-depth gene ontology and protein family analyses on the identified proteins and discussed comparisons against similar proteome data sets acquired for the Southeast Asian liver fluke *O. viverrini* and the Chinese liver fluke *C. sinensis*.

**Discussion:**

The information from this study will form a biologically relevant data set of *O. felineus* proteins that could be used to develop diagnostic and therapeutic tools to manage the human cost of *O. felineus* infection and its associated comorbidities.

## Introduction

Liver fluke infections caused by *Opisthorchis felineus* and *Opisthorchis viverrini* (opisthorchiasis) or *Clonorchis sinensis* (clonorchiasis) are estimated to currently infect approximately 45 million individuals across the globe (Saijuntha et al., 2021). *O. viverrini* and *C. sinensis* infections are limited to Southeast Asian countries and, although infections caused by *O. felineus* are typically endemic to the river plains of Western Siberia and Eastern Europe, outbreaks have been documented further west in areas as far as the Iberian Peninsula. Furthermore, it is estimated that more than 1.6 million people could be infected with these parasites (Petney et al., 2013; Pakharukova and Mordvinov, 2016; Fedorova et al., 2020). The fluke life cycle begins when fertilized eggs are released into freshwater bodies by the definitive hosts through defecation. When mature eggs are ingested by snails, they undergo transformative and reproductive changes to form free-swimming cercariae, which are shed into the water once more. Cercariae penetrate the skin of their second intermediate host, fish, where they encyst in the surface muscle or subcutaneous fat and become metacercariae (Pakharukova and Mordvinov, 2016; Yurlova et al., 2017). Definitive hosts (i.e., humans and other carnivorous mammals) get infected when eating raw or undercooked fish, and juvenile flukes excyst from metacercariae in the duodenum and ascend through the ampulla of Vater into the bile ducts, where they develop into adults (Kaewpitoon et al., 2008).

Infections with these liver flukes are typically characterized by inflammation, eosinophilia, and hepatobiliary injury and tissue remodeling. Furthermore, these trematode parasites have lifespans in excess of 10 years, resulting in chronic exacerbations of pathology at the infection site (Sripa et al., 2018). Indeed, *O. viverrini* and *C. sinensis* have been recognized as group 1 biological carcinogens and as definitive causes of bile duct cancer, or cholangiocarcinoma (CCA) (IARC, 2012). In the Republic of Korea, where *C. sinensi*s infections are endemic, a single-center study found that *C. sinensis*-associated CCA constituted 25.9% of CCA cases, and the median overall survival rate was found to be 12.7 months (Chang et al., 2021).

The exact mechanistic process leading from fluke establishment to malignancy is multifactorial. There is consensus that the combination of mechanical damage from fluke attachment and feeding, in addition exposure to fluke excretory/secretory (ES) products over a long period of time leads to chronic hepatobiliary perturbations and inflammation (IARC, 2012; Alsaleh et al., 2019). This causes oxidative stress and induces DNA damage, gene mutation, and dysregulated cell growth, in the form of goblet cell metaplasia, adenomatous hyperplasia, and epithelial hyperplasia, which sets the stage for carcinogenesis (Sripa et al., 2007; IARC, 2012; Alsaleh et al., 2019).

Despite sharing similar pathophysiology with *O. viverrini* and *C. sinensis*, *O. felineus* has not been recognized as a group 1 biological carcinogen at the time of the last review due to insufficient evidence (Pakharukova et al., 2019). However, since the last review by the International Agency for Research on Cancer (IARC) in 2012, several studies have been published on its contribution to CCA risk in humans and its carcinogenic potential in laboratory animals (Fedorova et al., 2017; Pakharukova et al., 2019; Mordvinov et al., 2021; Fedorova et al., 2022). A recent case–control study in Western Siberia found that individuals with a positive diagnosis by fecal egg microscopy and/or serum immunoglobulin M (IgM) or G (IgG) enzyme-linked immunosorbent assay (ELISA) had a significantly higher risk of developing CCA than non-infected individuals (Fedorova et al., 2022). This elevated risk was also found in patients who had records of a current or past infection with *O. felineus* (Fedorova et al., 2020; Fedorova et al., 2022). In addition, an allotransplantable CCA cell line (CCA-OF) was generated from CCA tissue induced in Syrian hamsters via *O. felineus* infection and dimethylnitrosamine administration (Pakharukova et al., 2019). Several biomarkers associated with both human CCA- and *O. viverrini*-associated experimental CCA were also expressed in the CCA–OF cell line (Korita et al., 2010; Hongsrichan et al., 2013; Khoontawad et al., 2014; Mordvinov et al., 2021). Despite its medical importance, contribution to cancer risk and the prevalence of infection, the different proteomes of *O. felineus* have not been extensively studied. In this study, we assembled an RNAseq transcriptome of the *O. felineus* adult-stage fluke, which was then used to search and identify proteins from the tandem mass spectrometric analysis of gel-fractionated *O. felineus* tegument, in addition to ES and 15K-extracellular vesicle (EV) extracts. We further characterized the putative functions of the identified proteins and described the notable proteins that may be important for identifying biomarkers of infection, developing novel therapeutics, and improving our understanding of liver fluke pathology and biology.

## Materials and methods

### *O. felineus* animal infections and fluke collection

All procedures with laboratory animals were conducted in accordance with guidelines for animal care. The experimental protocol was approved by the Institutional Animal Care and Use Committee (IACUC) in Siberian State Medical University, Tomsk (application number 06/2021, dated 06/07/2021). 4-week-old golden Syrian hamsters (*Mesocricetus auratus*) were obtained from the SPF-Vivarium of the Institute of Cytology and Genetics, ICG SB RAS (Novosibirsk, Russia).

The metacercariae for the induction of infection in hamsters were isolated from infected river fish (e.g., *Leuciscus leuciscus, Rutilus rutilus*) collected in Tomsk region, Russia. The muscular and subcutaneous tissues of the fish were gathered and digested with pepsin–hydrochloric acid (HCl) solution for 18 hours at 37°C. The viable metacercariae were washed with normal saline solution (0.9% sodium chloride) and counted using microscopy. Fifty (50) viable metacercariae were administered intragastrically to hamsters in 0.5 mL of normal saline solution, as previously described (Pershina et al., 2015).

After 8 weeks of *O. felineus* infection, all animals were euthanized by way of carbon dioxide (CO_2_) asphyxia. The livers were extracted, weighed, and placed inside a sterile Petri dish filled with normal saline solution. They were then dissected and adult flukes were collected, washed with normal saline, and placed into a new Petri dish containing 10 mL of wash buffer [1× phosphate-buffered saline (PBS), containing 2× antibiotic (100 U/mL penicillin–streptomycin)] for 30 minutes at 37°C.

### *O. felineus* fluke culture and excretory/secretory product collection

The washed flukes were transferred into a new Petri dish with 10 mL of preconditioning cultivation medium [Roswell Park Memorial Institute (RPMI) 1640, media, containing 2× penicillin–streptomycin)] and incubated at 37°C in a 5% CO_2_ atmosphere for 2 hours. Finally, the flukes were placed into wells of a six-well culture plates (50 flukes per well) filled with culture medium [RPMI 1640, containing 1× penicillin–streptomycin, 10 g/L glucose, 2 g/L sodium bicarbonate, and 1 μM protease inhibitor (E64)] at 37°C in a 5% CO_2_ atmosphere. The supernatant containing *O. felineus* ES products (*Of*ES) and those containing the adult worms from different wells were pooled so that three technical replicates for each sample were analyzed. The *Of*ES was collected daily during the first 3 days of cultivation and replaced with fresh complete medium. The collected *Of*ES was centrifuged at 2,090 *g* for 15 minutes to pellet the eggs and the supernatant was stored at −80°C until use. At the end of cultivation, the adult flukes were collected, washed with 1 × PBS and stored in 1-mL cryotubes at −80°C for RNA extraction. Prior to downstream processing, all *Of*ES aliquots were thawed, pooled, and purified, as described previously (Sotillo et al., 2016). In brief, *Of*ES were differentially centrifuged at 500 *g*, 2,000 g and 4,000 g at 4°C for 20 minutes each. The resulting supernatant was then concentrated using a 10-kDa Amicon^®^ spin concentrator (Merck Millipore, USA) in accordance with the manufacturer’s instructions.

### RNA extraction and sequencing

A total of 50 adult *O. felineus* flukes were mechanically homogenized in 1 mL of TRI Reagent^®^ (Sigma-Aldrich, USA) and extracted as per the manufacturer’s instructions. Two milligrams (2 mg) of RNA was resuspended in 20 mL of diethyl pyrocarbonate (DEPC)-treated H_2_O and submitted for RNA sequencing at the Australian Genome Research Facility.

### Transcriptome generation and assessment

The PacBio Iso-Seq analysis pipeline v3 was used to generate the initial contig set. To generate the peptide sequences, we followed Trinity *de novo* assembly best practices (Grabherr et al., 2011) and reduced the number of contigs containing small repetitive sequences using the Cluster Database at High Identity with Tolerance (CD-HIT) program (Fu et al., 2012). To integrate our transcriptome assembly with the existing WormBase transcriptome (available from https://ftp.ebi.ac.uk/pub/databases/wormbase/parasite/releases/WBPS18/species/opisthorchis_felineus/PRJNA413383/opisthorchis_felineus.PRJNA413383.WBPS18.CDS_transcripts.fa.gz), we combined the nucleotide files, performed basic quality control (QC) methods, as described previously (Thang et al., 2019), ran CD-HIT, removed all duplicate sequences, and followed Trinity best practices to generate peptide files. Finally, the completeness of the transcriptomes were assessed with BUSCO against Metazoa Odb10 (Simao et al., 2015). All analysis steps are available at https://github.com/mattmattmattmatt/Ofelineus.

### Tegument extraction

To enhance the solubilization of membrane proteins and deplete water-soluble cytosolic proteins, the *O. felineus* tegument was solubilized sequentially in three stages with buffers of increasing strength, in accordance with the previously published method (Braschi et al., 2006; Mulvenna et al., 2010), with each stage treated as its own extract for downstream analysis. The buffers were added to the sample pellet, vortexed for 2 minutes and incubated at the indicated temperature for each buffer for 20 minutes. At each stage, samples were extracted three times with a centrifugation of 100,000 *g* for 1 hour in between to pellet the insoluble material and collect supernatants, which were pooled in accordance with the extraction stage. The buffer composition, extract order, and incubation temperature used was as follows: TEG1—40 mM tris, at pH 7.4 at 4°C; TEG2—5 M urea in 40 mM tris, at pH 7.4 at room temperature; and, TEG3—0.1% SDS. The proteins from the pooled supernatants were precipitated with nine volumes of methanol at −20°C.

### 15K-EV isolation

The 15K-EVs were enriched from a separate aliquot of *Of*ES that was previously concentrated with a 10-kDa centrifugal concentrator (Merck Millipore, USA). The 15K-EVs were pelleted at 15,000 *g* for 1 hour at 4°C, as previously described (Cwiklinski et al., 2015), and further washed with PBS three times before storage at −80°C until use.

### SDS-PAGE gel and in-gel digestion

Proteome extracts were decomplexified using an in-gel digestion protocol, with each replicate yielding four gel slices. Up to 20 µg of protein for each of the three replicates from each *O. felineus* sample was resuspended in a glycerol-based loading buffer and incubated at 95°C for 5 minutes. The resuspended protein and a vehicle control were loaded into 4%–20% TruPAGE™ precast polyacrylamide gels (Sigma-Aldrich, USA) and run at 100 V in a tris–glycine 1% SDS running buffer until the dye front had traveled approximately 2 cm. Gels were stained with 0.02% Coomassie blue for 10 minutes and destained with destaining buffer (comprising 10% acetic acid, 50% methanol, and 40% H_2_O) twice for 45 minutes each. Gel lanes containing the proteins for each *O. felineus* fraction were sliced into four bands that were approximately 2 mm × 10 mm in size. A single control band was collected from the same area from the lane with the vehicle control. Each gel slice replicate was prepared, processed, and acquired on the mass spectrometer as a separate sample.

The gel slices were further destained in 200 µL of 50% volume-to-volume (v/v) acetonitrile and in 200 mM ammonium bicarbonate buffer at 37°C for 45 minutes twice, with fresh buffer added each time. The gel slices were dried thoroughly in a vacuum centrifuge before being incubated in 100 µL of 20 mM dithiothreitol and in 25 mM ammonium bicarbonate buffer at 65°C for 1 hour. The gel slices were then incubated in 100 µL of 50 mM iodoacetamide and 25 mM ammonium bicarbonate at 37°C for 40 minutes. The gel slices were washed in 200 µL of 25 mM ammonium bicarbonate at 37°C for 15 minutes and dried again in a vacuum centrifuge. Mass spectrometry (MS)-grade trypsin protease (Pierce™ Thermo Fisher, USA) stocks were resuspended in 50 mM acetic acid at a concentration of 1 mg/mL. The slices from each gel band were incubated with 0.5 µg of trypsin stock diluted in 100 µL of 100 mM ammonium bicarbonate at 37°C for approximately 18 hours. The tryptic peptides were extracted from gel slices three times using 50 µL of 50% acetonitrile and 0.1% trifluoroacetic (TFA) solution incubated at 37°C for 15 minutes each time. The tryptic peptides were dried in a vacuum centrifuge before further downstream processing.

### Solid-phase sample clean-up

Solid-phase stage tips were prepared by stacking three layers of Empore™ Octadecyl C18 (Supelco/Sigma-Aldrich, USA) disc punches in a 200-µL pipette tip. All the centrifugation steps were performed at 1,000 g for 1 minute–3 minutes, as required. The stage tips were supported over a 2-mL tube and washed via centrifugation with 100 µL of methanol and 100 µL of 0.2% TFA. The dried tryptic peptides, extracted from the gel slices, were resuspended in 0.2% TFA and loaded onto the stage tip over two rounds of centrifugation. The bound peptides were washed three times with 200 µL of 0.2% TFA, before a final elution step with 100 µL of 60% acetonitrile 0.2% formic acid was carried out. The eluted peptides were dried in a vacuum centrifuge concentrator and submitted for mass spectrometric analysis.

### Data acquisition

All samples were analyzed using a NanoLC 415 1D (Eksigent, USA) liquid chromatography system running on water with 0.1% (v/v) formic acid in water (solvent A) and 0.1% (v/v) formic acid in 100% high-performance liquid chromatography (HPLC)-grade acetonitrile (solvent B) coupled to a TripleTOF^®^ 5600 (SCIEX, USA) mass spectrometer running the Analyst^®^ TF 1.8.1 software (SCIEX, USA) at UQCCR (Brisbane, Australia). Each previously processed gel slice replicate was acquired on the mass spectrometer as a separate sample. The digested peptides from all replicates of the tegument fractions and two replicates each from the 15K-EV fractions were first loaded on a ChromXP 3C18-CL, 0.1 mm × 10 mm, 5-μm, 120-Å trap column (Eksigent, USA) under 10 μL/minute of solvent A for 10 minutes and separated on a ChromXP C18, 0.3 mm × 150 mm, 3-μm, 120-Å column (Eksigent, USA), with a linear gradient of 3%–32% solvent B over 45 minutes at a microflow rate of 5 μL/minute. The mass spectrometer experimental parameters were: curtain gas = 30, ion source gas 1 = 25, ion source gas 2 = 20, IonSpray voltage floating = 5,500V, and turbo heater temperature = 250°C. The ions were collected and analyzed with 250 ms time-of-flight mass spectrometry (TOF MS) followed by 23 experiments of 100 ms product ion data-dependent acquisition scans on product ions with an intensity greater than 100 counts and a charge state between +2 and +4. The mass windows were 350–1,800 m/z and 100–2,000 m/z for the TOF MS and product ion scans, respectively.

The digested peptides from all replicates of the ES fractions and one replicate from each of the 15K-EV fractions were first loaded on a ChromXP 3C18-CL, 0.1 mm × 10 mm, 5-μm, 120-Å trap column under 3 μL/minute of solvent A for 15 minutes and separated on a Eksigent ChromXP 3C18CL, 0.3 × 150 mm, 3-μm, 120-Å column, with a linear gradient of 4.8%–40% solvent B over 85 minutes at nanoflow rates of 0.25 μL/minute. The mass spectrometer experimental parameters were: curtain gas = 25, ion source gas 1 = 20, IonSpray voltage floating = 2,600V, and turbo heater temperature = 150°C. Ions were collected and analyzed with 200 ms TOF MS, followed by 45 experiments of 50 ms product ion information-dependent acquisition (IDA) scans on product ions with an intensity greater than 75 counts and a charge state between +2 and +5. The mass windows were 300–1,800 m/z and 100–2,000 m/z for the TOF MS and product ion scans, respectively.

### Database search and protein identification

The acquired mass spectrometry data files for each gel replicate were pooled according to its parent proteome and searched against the RNAseq-based *O. felineus* protein library generated in-house appended with the *Mesocricetus auratus* (golden/Syrian hamster) proteome from UniProt (UP000189706, retrieved 09/06/2023) and 381 sequences from a universal protein contaminant library (Frankenfield et al., 2022) using ProteinPilot 5.0.1 software (SCIEX, USA). Searches were conducted using the Paragon method, with the following settings in place: sample type = identification; cys alkylation = iodoacetamide, with other cys mods possible; digestion = trypsin; instrument = TripleTOF 5600;, ID focus = biological modifications; search effort = thorough ID; detected protein threshold ≥ 1.3 (95.0%); and run false-discovery rate analysis = yes. The search results were further filtered to exclude proteins with less than two peptides detected.

### Bioinformatics analysis of proteins

The gene ontology (GO) and protein family domain searches were carried out on the filtered protein lists using the Blast2GO v6.0.3 (Biobam) (Gotz et al., 2008) and HMMER 3.3.1 software (Eddy, 2011), respectively. The protein Basic Local Alignment Search Tool (BLAST) searches for GO annotations utilized the non-redundant National Center for Biotechnology Information (NCBI) database (Sayers et al., 2023) configured to a BLAST expectation value of 1.0E-3, word size of 6 and a high-scoring segment pair length cutoff value of 33. The HMMER software identified Pfam families and domains (Sayers et al., 2023) at a threshold of *p* < 0.01. REVIGO (Supek et al., 2011) was used to map the frequency of GO terms relative to the entire UniProt-to-GO database (Huntley et al., 2015) and to visualize and scale GO terms in the form of a two-dimensional semantic score scatter plot.

## Results

### Transcriptome database assembly

The PacBio Iso-Seq analysis pipeline v3 generated 87,553 initial contigs. Following Trinity best practices, we generated 13,028 final peptides, representing 4.66 Mb of amino acid sequences. To try to improve the existing WormBase transcriptome assembly we combined our long-read data with the existing assembly to generate a newly merged transcriptome assembly.

We input both the final PacBio-only and the merged assembly to BUSCO to benchmark and compare their performance with that of the Metazoan_Odb10 and the PacBio-only assembly; the Metazoan_Odb10 and the PacBio-only assembly captured 72.3% complete BUSCOs and 6.6% fragmented BUSCOs compared with the merged assembly, which captured 81.3% complete BUSCOs and 2.7% fragmented BUSCOs (**Supplementary Figure S1**). The merged assembly represents a substantial improvement over the current WormBase transcriptome assembly (standalone results not shown), with the merged assembly capturing 38 additional complete BUSCOs (81.3% complete in the merged vs. 77.2% complete in WormBase) and also reducing the number of missing BUSCOs by 29 (16.0% missing in the merged vs. 19.1% missing in WormBase). The final merged assembly contains 32,600 final protein sequences. All the transcriptomes are available at https://github.com/mattmattmattmatt/Ofelineus.

### Protein identification with mass spectrometry

**Figure 1:**
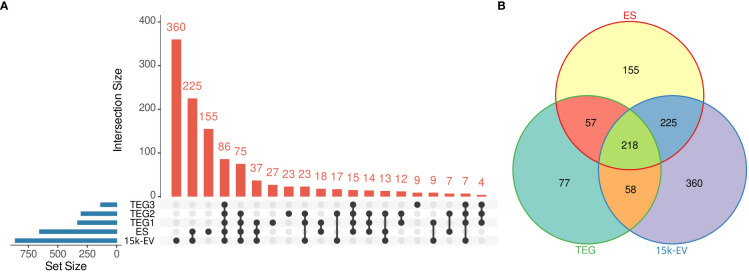
Summary of identified proteins from the respective adult *Opisthorchis felineus* proteomes. The number of unique and intersecting or shared proteins across each analyzed fraction was visualized on an UpSet plot. The blue bars represent the total number of proteins from each fraction, the dots or connected dots represent fractions within an intersection, and the red bars indicate the number of proteins in each intersection. **(A)** All unique proteins from the three tegument fractions were pooled and visualized on a Venn diagram against the whole excretory/secretory and microvesicular proteomes **(B)**.

The largest number of *O. felineus* proteins was identified in the 15K-EV extract, followed by the total ES fraction, with 861 and 655 proteins identified, respectively (**Figure 1A**). A total of 410 unique proteins were identified across the entire tegument protein compartment, with 331, 302, and 135 proteins identified in TEG1, TEG2, and TEG3 samples, respectively (**Figure 1A**). A total of 360, 155, and 77 proteins were uniquely identified in the 15K-EV extract, ES, and tegument, and 218 shared proteins were identified across all protein compartments (**Figure 1B**).

### Gene ontology analysis

**Figure 2:**
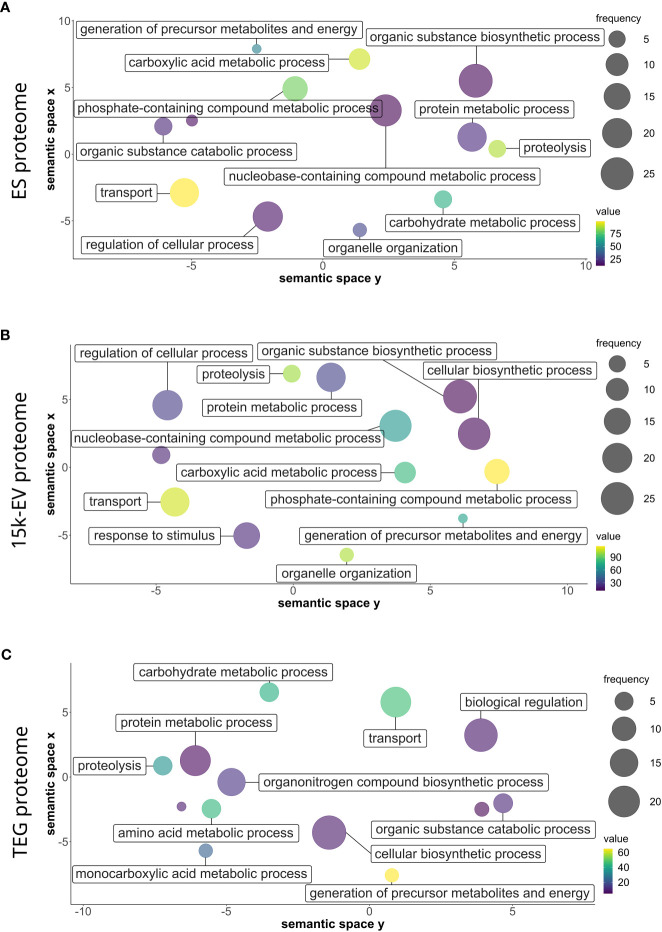
Gene ontology of biological processes of the respective adult *Opisthorchis felineus* proteomes. Proteins from the *O. felineus* whole excretory/secretory **(A)**, 15K-EV **(B)**, and tegument **(C)** proteomes were analyzed and annotated using Blast2GO to identify gene ontologies (GO). GO terms and their respective node score results from Blast2GO were plotted and visualized using REVIGO. The circle size indicates the relative frequency of the respective GO term relative to the entire UniProt gene ontology mapping database, in which a higher frequency indicates a more general and frequently occurring GO term. The color scale represents the node score, which is calculated based on the number of identified sequences and the strength of their association with the GO term, for which a larger number indicates an overall enrichment. Semantically similar GO terms are clustered more closely on the plot.

The gene ontology analysis of all identified protein sequences was conducted using Blast2GO to catalog the attributes and descriptions of sequences detected in each proteome compartment. The analyzed proteins with annotated GO terms were organized into broad categories of biological processes, cellular components, and molecular functions. Parent–child GO term redundancy was removed and results were visualized using REVIGO. Although only a small number of biological processes with relatively high node scores (i.e., “proteolysis”, and transport-related ontologies) were shared across the ES, 15K-EV, and TEG proteomes (**Figure 2**), some other terms were represented differently in each proteome. Notably, the functional group of “response to stimulus” was uniquely enriched in the 15K-EV fraction, whereas “amino acid metabolic process”, “monocarboxylic acid metabolic process”, “biological regulation”, and “organonitrogen compound biosynthetic process” were uniquely enriched in the TEG fraction (**Figure 2**). The proteins associated with the “cytoplasm” cellular component were enriched in the ES proteome (**Supplementary Figure S2**), whereas proteins involved with the terms “protein-containing complex” and “supramolecular fiber” were enriched only in the TEG proteome (**Supplementary Figure S2**). Different molecular function terms with high node scores, such as “metal ion binding”, “transferase activity”, and “oxidoreductase activity”, were conserved across all three proteomes. Interestingly, the term “hydrolase activity” was highly enriched in the TEG proteome (**Supplementary Figure S3**), whereas the proteins involved with “peptidase activity” were enriched only in the ES proteome (**Supplementary Figure S3**).

### Identified shared proteins

The protein descriptions for the unique and shared individual sequences initially identified across all three proteome compartments were extracted from the GO analysis to identify the possible proteins of interest. The shared protein sequences were further matched to their respective “unused protein scores”, which were derived from the mass spectrometry database search to prioritize proteins identified with the most confidence. Among the proteins shared across all three proteome compartments, the five proteins with the highest “unused protein score” in the ES proteome were described as being a basement membrane-specific heparan sulfate proteoglycan core protein, a GPI-anchored surface glycoprotein, TRPM8 channel-associated factor 2, and also two unidentified proteins (**Supplementary Table S1**). For the 15K-EV proteome, the top five highest-scoring proteins were described as being calpain, variant 2, taurocyamine kinase, a putative extracellular matrix glycoprotein, alpha-actinin (sacromeric), and an unidentified protein (**Supplementary Table S2**). Last, present within the description of the top five highest-scoring proteins in the TEG proteome were taurocyamine kinase, a heat shock cognate 71-kDa protein, spectrin alpha chain non-erythrocytic, glutathione *S*-transferase, and another unidentified protein (**Supplementary Table S3**).

### Protein family analysis of *O. felineus* and *C. sinensis* proteomes

**Figure 3:**
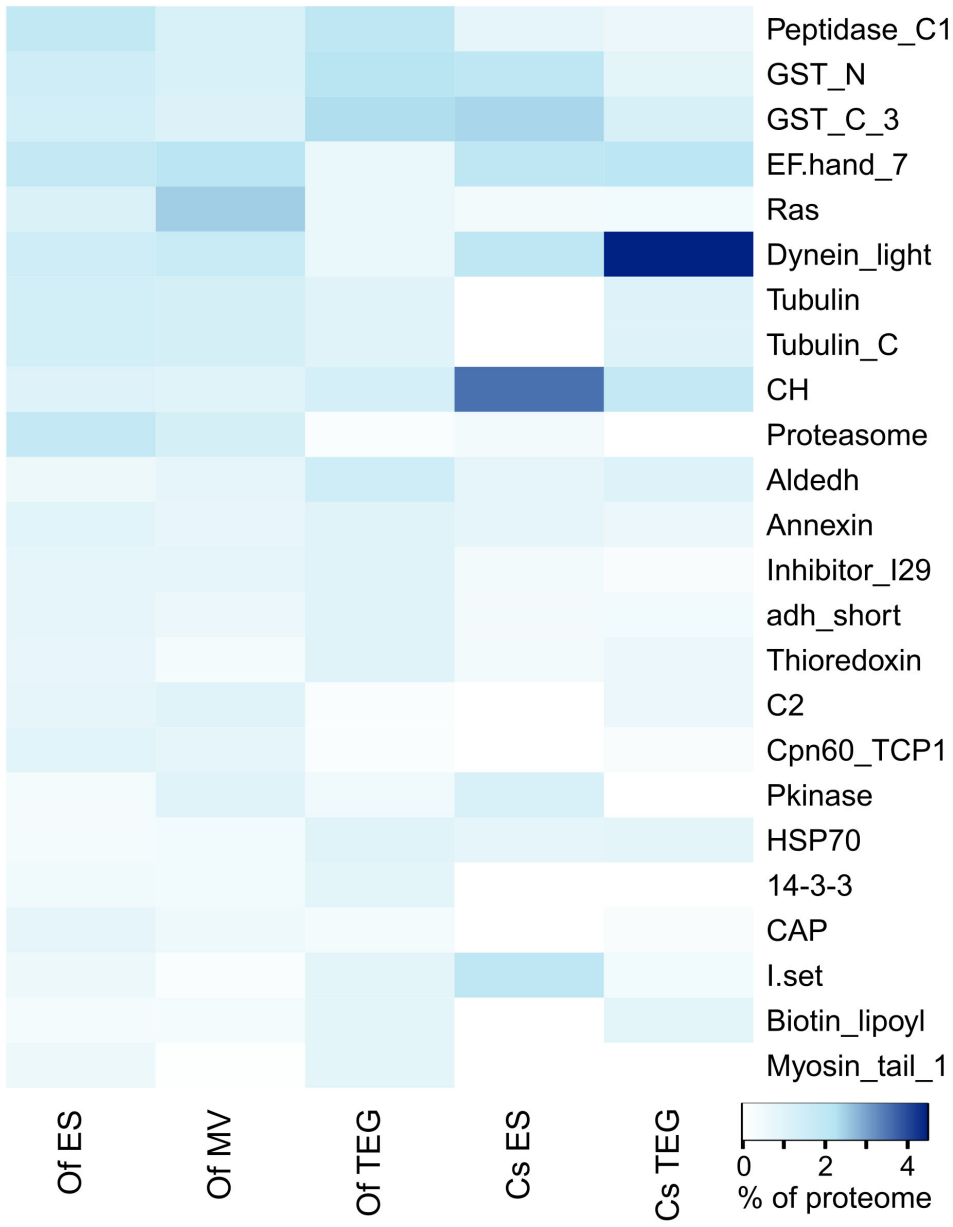
Pfam analysis of proteins from the respective adult *Opisthorchis felineus* proteomes. Top 20 protein family and domain (Pfam) counts from each proteome visualized on a heatmap. The color scale represents the counts of the respective protein families and domains matched to the Pfam database from the proteins detected and identified in each proteome.

A protein domain analysis using Pfam was applied to the identified proteins in each of the three *O. felineus* protein compartments under investigation (**Supplementary Table S4**). The top 15 most abundant Pfam terms by proportion of all Pfam counts in each of the ES, 15K-EV, and TEG proteomes were selected and shown in a heat map. The heat map also included the top 15 most abundant Pfam terms from an additional analysis applied to protein sequences identified from *C. sinensis* using mass spectrometry in a previous study (Shi et al., 2020) (**Figure 3**). Three protein families and domains were found to be enriched across all three *O. felineus* proteomes (the papain family cysteine proteases and two glutathione *S*-transferase domains). Five protein domains and families among the top 15 Pfam terms (proteasome subunit, EF-hand 7, dynein light chain type 1, TCP-1/cpn60 chaperonin, and C2) were noticeably enriched in the ES and 15K-EV proteome, as compared with the TEG proteome (**Figure 3**). The biotin lipoyl domain, Hsp70, and aldehyde dehydrogenase family were enriched in the TEG proteome compared with the ES and 15K-EV proteomes. Interestingly, the percentage of proteasome domain counts was found to be more than seven times higher in the ES and 15K-EV proteomes than it was in the TEG proteome.

The analysis of the previously published *C. sinensis* proteome showed that four families and domains were enriched across both the TEG and ES proteomes (calponin homolog, glutathione *S*-transferase C-terminal, dynein light chain, and EF hand 7). Compared with the *O. felineus* TEG proteome, the dynein light chain family and EF hand 7 were notably enriched in the *C. sinensis* TEG proteome, but the opposite was observed for the peptidase C1-like family and the glutathione *S*-transferase N- and C-terminal domains. The top five most abundant Pfam terms by proportion of counts from the *C. sinensis* ES proteome (calponin homolog, dynein light chain, immunoglobulin I-set, and the N- and C-terminal domains of glutathione *S*-transferase) were more frequent, as compared with the *O. felineus* ES proteome. The peptidase C1-like family and proteasome and Ras domains, which were relatively enriched in the *O. felineus* ES proteome, were found to be constitute a lower proportion of the overall Pfam counts in the *C. sinensis* ES proteome. Furthermore, the tubulin and tubulin C-terminal domains, which were relatively enriched in the *O. felineus* ES, were distinctly absent in the *C. sinensis* ES.

### Protein family analysis of unique and shared proteins from *O. felineus* proteomes

Among the 218 proteins shared between all three *O. felineus* proteomes, the top five most abundant protein families and domains were the glutathione *S*-transferase C- and N-terminal domains, annexins, calponin homolog, and the aldehyde dehydrogenase family (**Supplementary Table S5**). The papain family cysteine protease was the most abundant protein family identified in the *O. felineus* ES proteome, with a further six families and domains (laminin N-terminal, common central domain of tyrosinase, prolyl oligopeptidase, collagen triple-helix repeat, C-terminal tandem repeated domain in type 4 procollagen, and the fasciclin domain) tied for second place (**Supplementary Table S5**). Within the top five protein families and domains identified from proteins unique to the 15K-EV proteome, the Ras family, EF hand 7, and protein kinase domains were notably enriched; these were followed to a lesser extent by the dynein light chain type 1 and C2 domains (**Supplementary Table S5**). For proteins uniquely identified in the TEG proteome, the top five protein families and domains were the glutathione *S*-transferase C- and N-terminal domains, papain family cysteine proteases, the LIM domain, and the cathepsin propeptide inhibitor domain (I29) (**Supplementary Table S5**).

## Discussion

Several studies have comprehensively examined and characterized the tegument and ES proteomes from *C. sinensis* (Zheng et al., 2013; Wang et al., 2014; Shi et al., 2020), *O. viverrini* (Boonmee et al., 2003; Mulvenna et al., 2010), and *O. felineus* (Kovner et al., 2022). Despite its health implications, only one previous study sought to characterize the secreted proteome of *O. felineus*; most other studies have been more limited in their experimental scope and identified only small numbers of proteins (Pomaznoy et al., 2013; L'Vova et al., 2014; Pomaznoy et al., 2016). The transcriptomes of the adult and metacercaria stages of *O. felineus* were comprehensively elucidated in a study that generated 12,665 protein-encoding transcripts with Illumina sequencing (Pomaznoy et al., 2016). An earlier study used Sanger-sequenced cDNA from adult *O. felineus* total mRNA to assemble 267 contigs for a bioinformatic comparison against pre-existing gene ontology and protein sequences from several related parasites (Pomaznoy et al., 2013). To our knowledge, a study from 2014 identifying just 37 ES proteins, and a more recent 2022 study by Kovner et al. that identified 126 ES proteins (Kovner et al., 2022), are the only reports to date that utilized experimental mass spectrometry to characterize *O. felineus* proteins at the host–parasite interface. In this current study, 32,600 protein-encoding transcripts were generated and 660, 864, and 414 proteins were identified from the total ES, enriched 15K-EVs, and sequentially solubilized tegumental proteomes from *O. felineus*, respectively. Although we have not proven the origin of the 15K-EVs isolated in our study, several studies have found their gastrodermal or tegumental origin in other liver flukes (Marcilla et al., 2012; Cwiklinski et al., 2015), hence the important overlap in protein identification between the tegumental and 15K-EV fractions. The proteins and isoforms extracted from UniProt using the *O. felineus* ES protein IDs previously published (Kovner et al., 2022) were compared against all the ES proteins identified in this current study using the BLAST function in the Blast2GO software. The BLAST comparison showed that only three proteins in the study by Kovner et al. (2022) did not show any alignment or matches, and that only another three proteins had a BLAST e-value higher than 1E-30, indicating that almost all *O. felineus* ES proteins in the study by Kovner et al. (2022) aligned strongly with the ES proteins identified in this study (**Supplementary Table S11**).

During an infection, the host is continually exposed to proteins that are secreted and excreted, and to proteins present within the fluke’s tegument. Indeed, proteins at the host–parasite interface are critical to parasite survival, modulation of host biological processes, and pathogenesis (Gadelha et al., 2015; Shi et al., 2020). As yet, there is a significant knowledge gap with regard to the composition and function of *O. felineus* proteins at the host–parasite interactome. The comprehensive acquisition and characterization of these proteins in this study could facilitate a better understanding of this parasite, and, based on recent evidence of the involvement of *O. felineus* in CCA, lead to the improved diagnosis and management of the disease and associated pathogenesis.

Proteases are continuously shown to be a significant component of helminth secretomes (Wang et al., 2019; Abuzeid et al., 2020; Shi et al., 2020). GO analyses of the *O. felineus* ES, 15K-EV, and TEG proteomes in this study assigned, respectively, 69 (19.3%), 74 (15.3%), and 33 (14%) sequences with the GO term “proteolysis”. Furthermore, this term received a much higher node score in the ES and 15K-EV proteomes than in the TEG proteome, suggesting a relative enrichment of proteases secreted and excreted by *O. felineus*. Under the GO term “proteolysis”, several cathepsins were identified from the different protein samples analyzed and assigned to their respective GO subgroup. Namely, under the term “cysteine-type peptidase activity”, cathepsin C was found uniquely in the ES proteome, whereas cathepsin L was found only in the ES and 15K-EV proteomes. Under the GO term “cysteine-type endopeptidase activity” or “cysteine-type peptidase activity”, cathepsin B-like cysteine proteinase was found across all three proteomes, but cathepsin B was identified only in the ES and TEG proteomes. Under the term “aspartic-type endopeptidase activity”, cathepsin D was also found across all three *O. felineus* proteomes. Unsurprisingly, the protein family “papain family cysteine proteases”, to which cathepsins B and L belong, was one of the most highly enriched families across all three *O. felineus* proteomes.

Cathepsins are essential for both host and helminth survival, facilitating numerous biological functions such as nutrient uptake, ion channel activity, and vesicular transport (Patel et al., 2018; Shi et al., 2020). However, their dysregulation has also been associated with the development of several diseases, including some forms of cancer, and also autoimmune and cardiovascular diseases (Patel et al., 2018). Notably, levels of cathepsin B have been shown to be increased in the tissues or serum of cancer patients diagnosed with CCA, thyroid cancer, or colorectal cancer (McKerrow et al., 2000; Srisomsap et al., 2002; Monsouvanh et al., 2014). Several mechanisms for cathepsin B’s contribution to cancer development were demonstrated with *in vitro* and *in vivo* models, and its ability to proteolytically interfere with the apoptotic pathway was proposed to be a significant aspect of its oncogenic potential (Aggarwal and Sloane, 2014). For *C. sinensis*, only cathepsin C was detected in the ES and only cathepsin B-like cysteine proteinases were identified in the tegument (Shi et al., 2020). This finding is also congruent with the lower relative abundance of papain family cysteine proteases among *C. sinensis* host–parasite interface proteins, as shown in the Pfam analysis (**Figure 3**). Cathepsin F was previously identified in the *O. felineus* ES proteome (Kovner et al., 2022) and in the first report of the *O. viverrini* ES proteome by Mulvenna et al. (2010), only cathepsins F and D were selected for specific detection and were identified through one and three peptides, respectively (Mulvenna et al., 2010). Mulvenna et al. (2010) also detected the calcium-dependent cysteine-type endopeptidase calpain in the *O. viverrini* tegument and ES proteome (Mulvenna et al., 2010). Calpain is involved in many biological functions in both the host and helminth, and has been shown to be involved in immune evasion, parasite mobility, and protection against blood clots during *Schistosoma mansoni* infections (Wang et al., 2017; Chaimon et al., 2019). Furthermore, *S. mansoni* calpain is currently undergoing vaccine clinical trials, and *Leishmania major* calpain was also recently evaluated and shown to be a promising target for a T-cell epitope vaccine (Panzner et al., 2021; Brakat et al., 2022). Calpain may be a potential vaccine or diagnostic target for *O. felineus*, as calpain catalytic domain-containing protein was detected in the ES by Kovner et al. (2022) (Kovner et al., 2022), and calpain, variant 2 was detected in all three proteomes in this study, but further investigation is needed in this regard.

Several sequences across the ES, 15K-EV, and TEG proteomes were identified as cystatin domain proteins, with cysteine protease inhibitors falling under the GO term “negative regulation of endopeptidase activity”. Previously identified in the ES products of *C. sinensis*, *O. viverrni, O. felineus*, and many other helminths, cystatins inhibit and regulate cysteine protease enzymatic activity and are utilized by parasites to assist them in evading the host immune response (Mulvenna et al., 2010; Stachyra et al., 2019; Shi et al., 2020; Brakat et al., 2022; Kovner et al., 2022). Cystatins can regulate the proteases involved in the processing of peptides for presentation on MHCII complexes and the proteolytic activation of TLRs, which are key in the innate immune response (Vray et al., 2002; Cooper and Eleftherianos, 2016; Dall and Brandstetter, 2016). In addition, cystatins can promote IFN-γ-dependent nitric oxide production by macrophages, which may further exacerbate tissue pathology and oncogenesis at sites of infection via oxidative damage (Vray et al., 2002).

Members of the thioredoxin family such as thioredoxin 1, thioredoxin reductase, and thioredoxin peroxidase, are ubiquitous proteins involved in maintaining cellular reduction and oxidation balance and protecting against oxidative stress (Arner and Holmgren, 2000; Jia et al., 2019). Thioredoxin peroxidase prevents hydrogen peroxide accumulation by reducing it to water via donating hydrogen and forming disulfide bonds between its free thiol groups. Thioredoxin 1 is able to reduce oxidized thiol groups on thioredoxin peroxidases and other oxidized proteins by donating hydrogen to disulfide bonds, returning them to their reduced state. Thioredoxin reductase in turn catalyzes the reduction of thioredoxin 1 using NADPH as an electron source (Yamawaki et al., 2003; Arner and Holmgren, 2006; Jia et al., 2019). In mammals, cestodes, and trematodes, the protein thioredoxin glutathione reductase functions as the electron transporter for both the thioredoxin-dependent system and another thiol-based redox system dependent on glutathione (Ross et al., 2012). In addition, thioredoxins have been shown to exhibit a broad range of functions in studies on individuals in both healthy and diseased states, including immune modulation, cell signaling, transcription regulation, chemoresistance, apoptosis inhibition, and the promotion of cancer cell growth (Arner and Holmgren, 2000; Jia et al., 2019; Gencheva and Arner, 2022). Several thioredoxin domain-containing proteins were previously identified in the ES of O*. felineus*, whereas thioredoxin peroxidase and unspecified thioredoxins were identified in both the tegument and ES products of *O. viverrini* (Mulvenna et al., 2010; Kovner et al., 2022). Thioredoxin 1 from *O. viverrini* was shown to have the ability to mitigate oxidative stress-induced expression of apoptosis and DNA damage genes and to reduce the apoptosis of human cholangiocytes (Matchimakul et al., 2015). Thioredoxin 1 and thioredoxin peroxidase were identified in the tegument but not in the ES products of *C. sinensis* (Shi et al., 2020). Thioredoxin glutathione reductase was not identified in *O. viverrini* or in *C. sinensis*. In this current study, unspecified thioredoxins and thioredoxin domain-containing proteins, in addition to thioredoxin glutathione reductase and thioredoxin peroxidase were detected and identified across all three *O. felineus* proteomes, but thioredoxin 1 was detected only in the TEG proteome.

Granulins have been previously identified in *C. sinensis* and *O. viverrini* ES products as growth promoters associated with cancer development (Smout et al., 2009; Mulvenna et al., 2010; Shi et al., 2020; Wang et al., 2021; Chaiyadet et al., 2022). Granulin-encoding mRNAs were also identified in the *O. felineus* transcriptome from this paper. However, these proteins were not detected in any *O. felineus* proteomes using mass spectrometry both in this study and that by Kovner et al. (2022). This is likely due to the small size of the granulins, which could have resulted in the proteins being washed through the spin concentrators during the preparation stage for the ES products (Smout et al., 2015).

Tetraspanins are membrane-spanning proteins that are expressed by multiple helminth parasites and their mammalian hosts (Drurey et al., 2020). *O. viverrini* tetraspanin expression was detected in the metacercariae and egg developmental stages and also in the tegument and extracellular vesicles of adult worms (Piratae et al., 2012; Chaiyadet et al., 2017; Chaiyadet et al., 2019). The vaccination of hamsters with the recombinant tetraspanins r*Ov*-TSP-2 and r*Ov*-TSP-3, followed by parasite challenge, resulted in significantly reduced fluke burdens and the stunting of surviving flukes recovered from vaccinated animals (Chaiyadet et al., 2019). Moreover, hamsters treated with a monoclonal IgM antibody raised against r*Ov*-TSP-2 developed significantly lower worm and egg burdens after *O. viverrini* infection challenge than did an isotype-matched control IgM (Phumrattanaprapin et al., 2021). In the current study, tetraspanin–CD63 receptors were detected in the ES and 15K-EV proteomes but not in the tegument, which is in contrast with what has been described in previous studies involving *O. viverrini*. An unspecified tetraspanin was also detected in the ES of *O.felineus* by Kovner et al. (2022). Comparing *O. felineus* tetraspanins detected via mass spectrometry against the published translated tetraspanin sequences from *O. viverrini* (*Ov*-TSP-1, *Ov*-TSP-2, and *Ov*-TSP-3) (Piratae et al., 2012; Chaiyadet et al., 2017), using the BLAST function of Blast2GO, revealed that the detected *O. felineus* tetraspanins had a similarity of 50% or less with the published *O. viverrini* tetraspanins (**Supplementary Table S12**). However, several homologs of the published *O. viverrini* tetraspanins were identified using a BLAST search of the *O. felineus* transcriptome generated in this study (**Supplementary Table S11**). Although *O. felineus* tetraspanins may indeed be promising vaccine candidates, further studies will need to be conducted to characterize and validate these proteins at the molecular level before testing for immunogenicity.

Annexins are a diverse superfamily comprising more than 1,000 proteins defined by the presence of a Ca^2+^-binding core domain sharing similar 70-amino-acid-long repeats (Moss and Morgan, 2004). Annexins are expressed by all eukaryotic cells and are associated with a large scope of cellular processes, ranging from cell membrane interactions and organization to maintaining the homeostasis of molecules such as glucose, cholesterol, calcium, and fibrin (Moss and Morgan, 2004; Schloer et al., 2018; Alvarez-Guaita et al., 2020; Mendez-Barbero et al., 2021). In the analysis by Kovner et al., a few unspecified annexins were identified in the ES of *O. felineus* (Kovner et al., 2022). In this study, annexins A6, A7, and A11 were identified across all three analyzed *O. felineus* proteomes. Comparatively, only annexins 9 and A13 were found in the tegument (but not in the ES from *O. viverrini*), and two unspecified annexins and annexin A7 were identified in the tegument of *C. sinensis*, but only the two unspecified annexins were detected in the ES products (Mulvenna et al., 2010; Shi et al., 2020). It appears that across the respective proteomic studies on these three liver flukes, annexins may be more enriched in the tegument proteomes than in the ES products. Previous studies have demonstrated that helminth annexins may play a role in parasite survival and establishment by attenuating the host processes, such as immune responses, which could be hostile to the parasite (Hofmann et al., 2010; He et al., 2014; Alvarez-Guaita et al., 2020). *C. sinensis* annexin B30 was shown to bind the human protease plasminogen and to stimulate the secretion of the anti-inflammatory cytokine IL-10 in splenocytes isolated from recombinant annexin B30-immunized rats (He et al., 2014). It was demonstrated that *Taenia solium* annexin B1 is able to bind to human eosinophils in a Ca^2+^-dependent manner, induce transient Ca^2+^ influx, and increase caspase-3 activity and apoptosis, thus providing further evidence for its role in evading host defense processes (Alvarez-Guaita et al., 2020).

This study is the first comprehensive high-throughput proteomic analysis of the ES, 15K-EV, and tegument proteomes of the parasitic helminth *O. felineus*. In addition, this study improves on the existing published literature concerning the *O. felineus* transcriptome ES proteome. This fluke’s secretome sheds light on the molecular events that govern the host–parasite interface in carcinogenic fluke infections and offers an important database for the future exploration of potential therapeutic and diagnostic antigen discovery efforts against *O. felineus* infection.

## Data availability statement

The datasets presented in this study can be found in online repositories. The names of the repository/repositories and accession number(s) can be found below: https://www.ebi.ac.uk/pride/archive/, PXD045798; https://www.ncbi.nlm.nih.gov/, PRJNA924086.

## Ethics statement

The animal study was approved by Institutional Animal Care and Use Committee (IACUC) in Siberian State Medical University, Tomsk (application No. 06/2021, dated 06/07/2021). The study was conducted in accordance with the local legislation and institutional requirements.

## Author contributions

YW, MP, AL, and JS contributed to experimental design. SR and BA assisted with mass spectrometry acquisition and method design. YW prepared and submitted mass spectrometry samples, analyzed the data, and drafted the manuscript. OF, VI, and EK collected and provided *O. felineus* material. MP and BT pre-processed samples and submitted material for RNA sequencing. MF analyzed RNA sequencing data. SR and BA acquired samples and collected mass spectrometry data. JS assisted with analysis of mass spectrometry results. AL and JS supervised the project. AL, MP, and OF secured funding for the project. All authors contributed to the article and approved the submitted version.

## Supplementary material

Supplementary Figure 1 BUSCO analysis of the adult *Opisthorchis felineus* transcriptome. The assembled transcripts were analyzed using BUSCO software against a metazoan gene list to quantitatively assess the completeness of the transcript database.

Supplementary Figure 2 Cellular component gene ontology from the respective adult *Opisthorchis felineus* proteomes. Proteins from the *O. felineus* whole-excretory/-secretory **(A)**, microvesicles **(B)**, and tegument **(C)** proteomes analyzed and annotated by Blast2GO to identify gene ontologies and node scores. The results were plotted and visualized using REVIGO. The circle size indicates the relative frequency of the GO term in the data and the color scale represents the node score. Semantically similar terms are clustered more closely.

Supplementary Figure 3 Molecular function gene ontology from the respective adult *Opisthorchis felineus* proteomes. Proteins from the *O. felineus* whole-excretory/-secretory **(A)**, microvesicle **(B)**, and tegument **(C)** proteomes analyzed and annotated using Blast2GO to identify gene ontologies and node scores. The results were plotted and visualized using REVIGO. The circle size indicates the relative frequency of the GO term in the data and the color scale represents the node score. Semantically similar terms are clustered more closely.
