# Supplementary figures and images for: Secreted and surface proteome and transcriptome of Opisthorchis felineus
Source: Front Parasitol. 2023 Oct 10;2:1195457. doi: 10.3389/fpara.2023.1195457 (PMC11732047; doi:10.3389/fpara.2023.1195457)

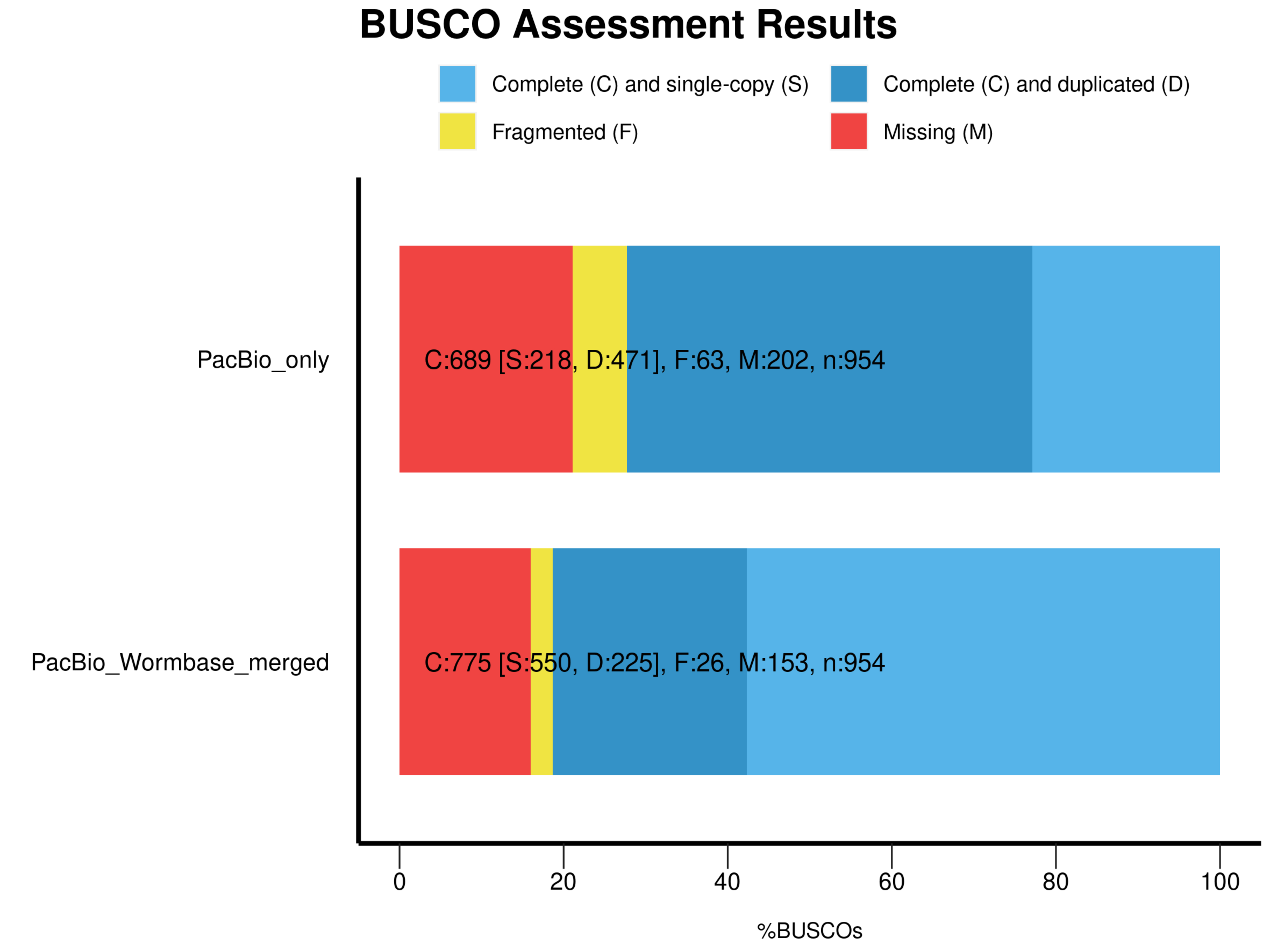

Supplement: Supplementary Figure 1 — BUSCO analysis of the adult Opisthorchis felineus transcriptome. The assembled transcripts were analyzed using BUSCO software against a metazoan gene list to quantitatively assess the completeness of the transcript database. [file Image_1.tiff]

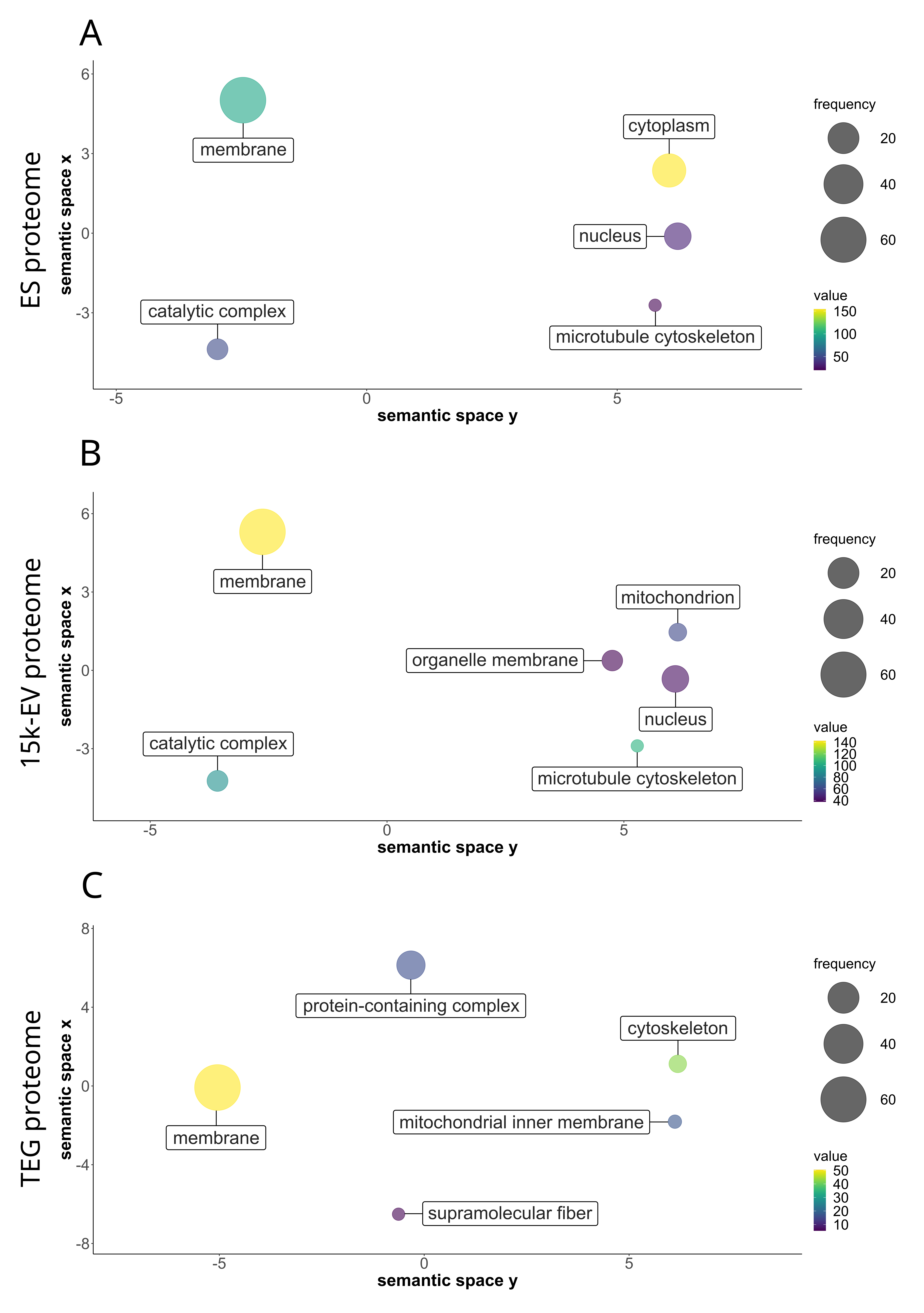

Supplement: Supplementary Figure 2 — Cellular component gene ontology from the respective adult Opisthorchis felineus proteomes. Proteins from the O. felineus whole-excretory/-secretory (A), microvesicles (B), and tegument (C) proteomes analyzed and annotated by Blast2GO to identify gene ontologies and node scores. The results were plotted and visualized using REVIGO. The circle size indicates the relative frequency of the GO term in the data and the color scale represents the node score. Semantically similar terms are clustered more closely. [file Image_2.tiff]

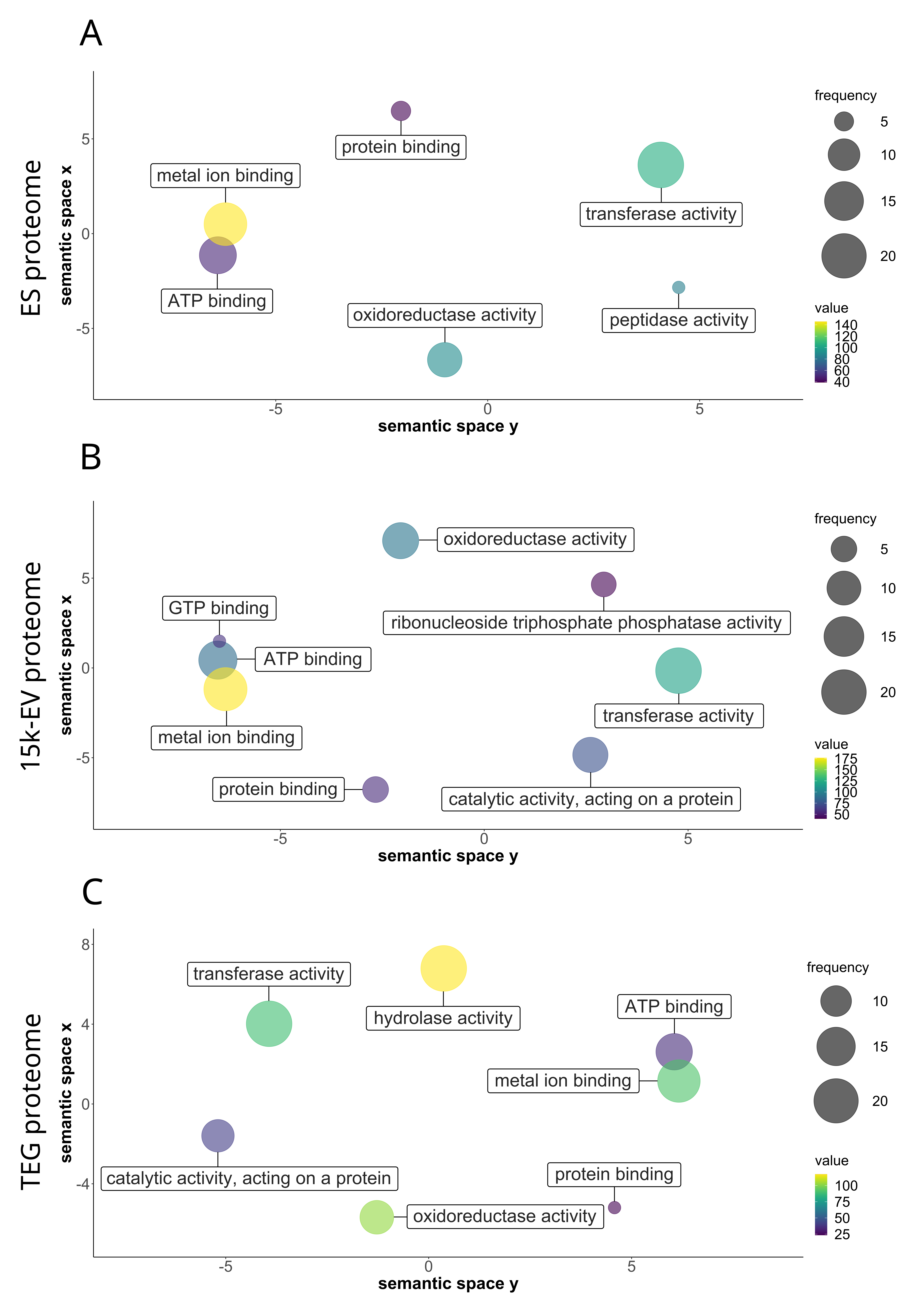

Supplement: Supplementary Figure 3 — Molecular function gene ontology from the respective adult Opisthorchis felineus proteomes. Proteins from the O. felineus whole-excretory/-secretory (A), microvesicle (B), and tegument (C) proteomes analyzed and annotated using Blast2GO to identify gene ontologies and node scores. The results were plotted and visualized using REVIGO. The circle size indicates the relative frequency of the GO term in the data and the color scale represents the node score. Semantically similar terms are clustered more closely. [file Image_3.tiff]
