# Supplementary material for: Secreted and surface proteome and transcriptome of Opisthorchis felineus
Source: Front Parasitol. 2023 Oct 10;2:1195457. doi: 10.3389/fpara.2023.1195457 (PMC11732047; doi:10.3389/fpara.2023.1195457)
Supplement: Supplementary file 16 [file DataSheet_1.pdf]

```
Percent identity summary
                    Percent identity (%)
Sequence    Description             Ov_tsp1     Ov_tsp2     Ov_tsp3
Of_RNA_HQ_transcript:34434.p1   Tetraspanin-CD63 receptor               21%     19%     19%
Of_RNA_HQ_transcript:13813.p1   Tetraspanin-CD63 receptor               17%     19%     17%
Of_RNA_HQ_transcript:44904.p1   Tetraspanin-CD63 receptor               15%     14%     19%


1. Of_RNA_HQ_transcript:34434.p1 against Ov_tsp1

Percent identity: 21%
NW Score: 6

Query  1    QTFEWSAIQYKNMCSLGSKILLSILNSIVIILGLILIIVGAIVAWGTHLILKLFEGPAQK  60
                         C    +ILL + N +V+++GL+++     V+          E  AQ
Sbjct  1    MMG----------CVQCLRILLVVFNFLVVLVGLVVLGFSVYVSQ---------EPEAQD  41

Query  61   YIESLGQDKTHIVKLAIREIGSMARPIGLLVFFLGLIILGIAIFGCVGATCNNKLCLKIY  120
             I + G    H V + I     M          +G I L  A+FGC GA   ++  L  Y
Sbjct  42   IIRASG----HYVAVQIALYALMG---------VGGITLITALFGCCGAYHESQCLLGAY  88

Query  121  VIILSVIVLVHILILIIHFSRPTLIMSPIKSELERYVKQYKSIQSG-EAASVFLSLLMTS  179
             IIL VI    +    + +     IM  ++ ++   V++Y  ++   E  S F+  +
Sbjct  89   FIILLVIFTSQVTGATLGYVFREEIMQHVEQQMFEGVEEYSMLRDQRENPSPFMDNIHRV  148

Query  180  LQCCGANGWEDFKQAKQMSKTDKFMQLEVNDLKFPLMCCKVNERFFPSDPENCIKNPDDK  239
            LQCCG NG+ D++                   + P+ CC   +        NC +
Sbjct  149  LQCCGVNGYTDYRD------------------RIPVTCCDRRK-------SNCNELQLTP  183

Query  240  NSNINNGCTKALEKYMVSTFNKILYGSLILLGINIALILLTVFALV                285
            +      GC    EKY     +K++   LI + I    I   +F++V
Sbjct  184  DEVYTEGCK---EKYKRFFKDKLIVFFLIAVSIAAFEIFCLLFSMVLCCAIRQYHSDYYG  240

Sbjct  241  VDYAIAT  247


2. Of_RNA_HQ_transcript:13813.p1 against Ov_tsp1

Percent identity: 17%
NW Score: -93

Query  1    TAVTSFLPKYFRTRLNWMVSRENEESSQQRRLSCCEKMLCNIPCRIVLFVINLISSIVAA  60
                                           + C + +      RI+L V N +  +V
Sbjct  1    M------------------------------MGCVQCL------RILLVVFNFLVVLVGL  24

Query  61   LLIGVGALMVWGNSVMEVVLMKVLFPLISAFTQKGDSEGIKQIVQRLMTTSAPIGYAIFG  120
            +++G    +                      +Q+ +++ I +     +     + YA+ G
Sbjct  25   VVLGFSVYV----------------------SQEPEAQDIIRASGHYVAVQIAL-YALMG  61

Query  121  VGLFVLIISAIGYIGACFNSKIAFKVYVSILGFLVIVLLAGIITYFAMKTKATDFVEDLF  180
            VG   LI +  G  GA   S+     Y  IL  +    + G    +  + +    VE
```

```
Sbjct  62   VGGITLITALFGCCGAYHESQCLLGAYFIILLVIFTSQVTGATLGYVFREEIMQHVEQQM  121

Query  181  LECVHNYLNMETNDGN-SLVVGFISPALGCCGLHNSSEFSSMRSNDTYGGKSYVDLKHPI  239
             E V  Y  +     N S  +  I   L CCG++  +++                 + P+
Sbjct  122  FEGVEEYSMLRDQRENPSPFMDNIHRVLQCCGVNGYTDYRD---------------RIPV  166

Query  240  ICCKMDEHYQLVYEDCPTGFTANNSNIYTP-CQEPLRKAF---LQYFDYLVFGIAGLMGL  295
             CC   +       +C          +YT  C+E  ++ F   L  F  +   IA
Sbjct  167  TCCDRRK------SNC-NELQLTPDEVYTEGCKEKYKRFFKDKLIVFFLIAVSIAAFEIF  219

Query  296  VLVLMCFTLCTIC--------VDIV     312
             L+      C I         VD
Sbjct  220  CLLFSMVLCCAIRQYHSDYYGVDYAIAT  247
```

3. Of_RNA_HQ_transcript:44904.p1 against Ov_tsp1

Percent identity: 15%
NW Score: -106

```
Query  1    MDAELYDLCSLWQFRARRNRWQKRIVL-WHTDVCPKPRCFKF------------------  41
            M   +  L  L             +VL +   V  +P
Sbjct  1    MMGCVQCLRILLVVFNFLVVLVGLVVLGFSVYVSQEPEAQDIIRASGHYVAVQIALYALM  60

Query  42   --------RSMISDNPSHHQYATLVGVLALAVMVCFS-------VYFAAKDKIADYVVKV  86
                     ++     ++H+   L+G   + ++V F+       + +  +++I  +V +
Sbjct  61   GVGGITLITALFGCCGAYHESQCLLGAYFIILLVIFTSQVTGATLGYVFREEIMQHVEQQ  120

Query  87   FARSVKEYKSMEANTVD-SLIVGLIQPPLRCCGVTSSADFVSLAATDNYGGQSYAGLTAP  145
                V+EY  +     + S  +  I   L+CCGV    D+                   P
Sbjct  121  MFEGVEEYSMLRDQRENPSPFMDNIHRVLQCCGVNGYTDYRD---------------RIP  165

Query  146  IPCCMMNDKYQITGAGCPASFTSANSYIDVGCKDPLKSKFIQYMNYVAYGLIGAFVILLL  205
            + CC   D+ +   + C     + +     GCK+   K  F   +       +      +
Sbjct  166  VTCC---DRRK---SNCNELQLTPDEVYTEGCKEKYKRFFKDKLIVFFLIAVSIAAFEIF  219

Query  206  VVLFTILTVC-----------ID---VV  219
             +LF+++  C           +D
Sbjct  220  CLLFSMVLCCAIRQYHSDYYGVDYAIAT  247
```

4. Of_RNA_HQ_transcript:34434.p1 against Ov_tsp2

Percent identity: 19%
NW Score: -32

```
Query  1    QTFEWSAIQYKNMCSLGSKILLSILNSIVIILGLILIIVGAIVAWGTHLILKLFEGPAQK  60
                  ++     C    +I+L ILN+ VI  G+ LI+VG++               A+
Sbjct  1    MV----SLSCGYRCL---QIILVILNTFVIACGVGLIVVGSL---------------AEV  38

Query  61   YIESLGQDKTHIVKLAIREIGSMARPIGLLVFFLGLIILGIAIFGCVGATCNNKLCLKIY  120
```

```
             +++ G+     +++ +           + +  LG +   I   G  GA   N   L  Y
Sbjct  39   SLKTFGESNETSIQIIV-----------IFIICLGCLTFLIGFLGFCGACLKNVCMLITY  87

Query  121  VIILSVIVLVHILILIIHFSRPTLIMSPIKSELERYVKQYKSIQSGEAASVFLSLLMTSL  180
             I+L V  +  ++  II       I   +   LE    +Y +    +     ++++ + L
Sbjct  88   AILLGVTAVAQVVCGIIGLVLRDKIPGLVNHNLEVLYTEYSA---NKDVQKLINVIQSEL  144

Query  181  QCCGANG-WEDFKQAKQMSKTDKFMQLEVNDLKFPLMCCKVNERFFPSDPENCIKNPDDK  239
            +CCGA G W +                                    S+PE+C ++P+
Sbjct  145  KCCGATGTWANPG----------------------------------SEPESC-RSPE--  167

Query  240  NSNINNGCTKALEKYMVSTFNKILYGSLILLGINIALILLT-----VFAL-------V  285
                 +GC   +E+++    N +  G  + +   I LI +T     V AL       V
Sbjct  168  GVVYKDGCVPKVEEFIQE--NMVAIGVCVFIFALIQLICMTFAICVVQALRKGEGETV  223
```

5. Of_RNA_HQ_transcript:13813.p1 against Ov_tsp2

Percent identity: 19%
NW Score: -119

```
Query  1    TAVTSFLPKYFRTRLNWMVSRENEESSQQRRLSCCEKMLCNIPCRIVLFVINLISSIVAA  60
                             MVS           LSC  + L     +I+L ++N
Sbjct  1                    MVS-----------LSCGYRCL-----QIILVILNTF-----V  22

Query  61   LLIGVGALMVWGNSVMEVVLMKVLFPLISAFTQKGDSEGIKQIVQRLMTTSAPIGYAIFG  120
            +  GVG ++V   S+ EV L          F +  +            T+   I   I
Sbjct  23   IACGVGLIVV--GSLAEVSL--------KTFGESNE------------TSIQIIVIFIIC  60

Query  121  VGLFVLIISAIGYIGACFNSKIAFKVYVSILGFLVIVLLAGIITYFAMKTKATDFVEDLF  180
            +G    +I  +G+ GAC  +      Y  +LG   +  +   I    ++ K    V
Sbjct  61   LGCLTFLIGFLGFCGACLKNVCMLITYAILLGVTAVAQVVCGIIGLVLRDKIPGLVNHN-  119

Query  181  LECVHNYLNMETNDGNSLVVGFISPALGCCGLHNSSEFSSMRSNDTYGGKSYVDLKHPII  240
            LE +  Y     N     ++   I   L CCG           +  T+          P
Sbjct  120  LEVL--YTEYSANKDVQKLINVIQSELKCCG-----------ATGTWANPG----SEPES  162

Query  241  CCKMDEHYQLVYEDCPTGFTANNSNIYTPCQEPLRKAFLQYFDYLVFGIAGLMGLVLVLM  300
            C   +    +VY+D                  P  + F+Q  + +  G+   +  ++ L+
Sbjct  163  CRSPEG---VVYKDGCV---------------PKVEEFIQE-NMVAIGVCVFIFALIQLI  203

Query  301  CFTLCTICV---------DIV  312
            C T   ICV         + V
Sbjct  204  CMTFA-ICVVQALRKGEGETV  223
```

6. Of_RNA_HQ_transcript:44904.p1 against Ov_tsp2

Percent identity: 14%
NW Score: -76

```
Query  1    MDAELYDLCSLWQFRARRNRWQKRIVLWHTDVCPKPRCFKFRSMISDNPSHHQYATLVGV  60
            M +       L       N +     +    V         ++    N +  Q   +  +
Sbjct  1    MVSLSCGYRCLQIILVILNTFVIACGVGLIVVGSLAEV-SLKTFGESNETSIQIIVIF-I  58

Query  61   LALAVMVCFSVYFAAKDKIADYVVKVFARSVKEYKSMEANTVDSLIVGLIQPPLRCCGVT  120
            + L  +     +          V  +   ++    +  A  V   I+GL+        V
Sbjct  59   ICLGCLTFLIGFLGFCGACLKNVCMLITYAILLGVTAVAQVVCG-IIGLVLRDKIPGLVN  117

Query  121  SSADFVSLAATDNYGGQSYAG-LTAPIPCCMMNDKYQITGAGCPASFTSANSYIDVGCKD  179
             + + +    + N   Q     + + + CC     +   G+   +  +        GC
Sbjct  118  HNLEVLYTEYSANKDVQKLINVIQSELKCCGATGTWANPGSEPESCRSPEGVVYKDGCV-  176

Query  180  PLKSKFIQYMNYVAYGLIGAFVILLLVVLFTILTVCI---------DVV  219
            P   +FIQ  N VA G+   F+  L+ ++     +C+         + V
Sbjct  177  PKVEEFIQE-NMVAIGVC-VFIFALIQLICMTFAICVVQALRKGEGETV  223
```

7. Of_RNA_HQ_transcript:34434.p1 against Ov_tsp3

Percent identity: 19%
NW Score: -23

```
Query  1    QTFEWSAIQYKNMCSLGSKILLSILNSIVIILGLILIIVGAIVAWGTHLILKLFEGPAQK  60
                  +  YK +     + +L + N +VI  G+ LI+VG+I      + LK +
Sbjct  1    MVS--LSCGYKCL-----QCMLVVFNVVVICCGIALIVVGSIA----QVQLKTY------  43

Query  61   YIESLGQDKTHIVKLAIREIGSMARPIGLLVFFLGLIILGIAIFGCVGATCNNKLCLKIY  120
                          L+  +   MA  I ++ F   L ++G   FG  GA   N  CL +Y
Sbjct  44   --------------LSSEDAQLMAFVIFIIAFGCFLTVVGS--FGFCGACKKNVCCLTMY  87

Query  121  VIILSVIVLVHILILIIHFSRPTLIMSPIKSELERYVKQYKSIQSGEAASVFLSLLMTSL  180
            +I L + +L  +   I  F    ++   +K  +++ + Q     + E +   + L+   L
Sbjct  88   IIFLVIFILGGVAAGIAGF----VLKDHVKEYVDKVLTQTYKTYNEEVSKKLIDLIQKDL  143

Query  181  QCCGANGWEDFKQAKQMSKTDKFMQLEVNDLKFPLMCCKVNERFFPSDPENCIKNPDDKN  240
             CCG +G                         +P    +V        P++C    D
Sbjct  144  GCCGPDG------------------------TWPPGLGQV--------PDSC---RDSSG  168

Query  241  SNINNGCTKALEKYMVSTFNKI-----LYGSLILLGINIALILLTVFAL---V  285
                 GC+ AL+K++      +     L+  L +L +  A+ +
Sbjct  169  LQYTQGCSAALDKFIEKNILAVALCVFLFAPLQILALVFAVCVCKAIQRGEDA  221
```

8. Of_RNA_HQ_transcript:13813.p1 against Ov_tsp3

Percent identity: 17%
NW Score: -94

```
Query  1    TAVTSFLPKYFRTRLNWMVSRENEESSQQRRLSCCEKMLCNIPCRIVLFVINLISSIVAA  60
                             MVS           LSC  K L    C +V+F + +I   +A
Sbjct  1                     MVS-----------LSCGYKCL---QCMLVVFNVVVICCGIAL  29
```

```
Query  61   LLIGVGALMVWGNSVMEVVLMKVLFPLISAFTQKGDSEGIKQIVQRLMTTSAPIGYAIFG  120
            +++G         S+ +V L          +    D++ +  ++             I
Sbjct  30   IVVG---------SIAQVQL--------KTYLSSEDAQLMAFVI------------FIIA  60

Query  121  VGLFVLIISAIGYIGACFNSKIAFKVYVSILGFLVIVLLAGIITYFAMKTKATDFVEDLF  180
             G F+ ++ + G+ GAC  +     +Y+  L   ++  +A  I  F +K    ++V+ +
Sbjct  61   FGCFLTVVGSFGFCGACKKNVCCLTMYIIFLVIFILGGVAAGIAGFVLKDHVKEYVDKVL  120

Query  181  LECVHNYLNMETNDGNSLVVGFISPALGCCGLHNSSEFSSMRSNDTYGGKSYVDLKHPII  240
             +    Y      + +  ++  I   LGCCG   +      +  D+    S   L++
Sbjct  121  TQTYKTY----NEEVSKKLIDLIQKDLGCCGPDGTWPPGLGQVPDSCRDSS--GLQYTQG  174

Query  241  CCKMDEHYQLVYEDCPTGFTANNSNIYTPCQEPLRKAFLQYFDYLVFGIAGLMGLVLVL-  299
            C    +            F   N      C               VF  A L  L LV
Sbjct  175  CSAALDK-----------FIEKNILAVALC---------------VFLFAPLQILALVFA  208

Query  300  MCFTLCTICVDIV  312
            +C        +
Sbjct  209  VCVCKAIQRGEDA  221
```

9. Of_RNA_HQ_transcript:44904.p1 against Ov_tsp3

Percent identity: 19%
NW Score: -102

```
Query  1    MDAELYDL----CSLWQFRARRNRWQKRIVLWHTDVCPKPRCF------KFRSMISDNPS  50
            M +         C L  F          +++  +    + + +      +  + +    +
Sbjct  1    MVSLSCGYKCLQCMLVVFNVVVICCGIALIVVGSIAQVQLKTYLSSEDAQLMAFVIFIIA  60

Query  51   HHQYATLVG-----------VLALAVMVCFSVYF------------AAKDKIADYVVKVF  87
               + T+VG           V  L + + F V F              KD + +YV KV
Sbjct  61   FGCFLTVVGSFGFCGACKKNVCCLTMYIIFLVIFILGGVAAGIAGFVLKDHVKEYVDKVL  120

Query  88   ARSVKEYKSMEANTVDSLIVGLIQPPLRCCGVTSSADFVSLAATDNYGGQSYAGLTAPIP  147
             ++ K Y       V   ++ LIQ  L CCG                 G    GL
Sbjct  121  TQTYKTYNE----EVSKKLIDLIQKDLGCCGPD---------------GTWPPGLGQVPD  161

Query  148  CCMMNDKYQITGAGCPASFTSANSYIDVGCKDPLKSKFIQYMNYVAYGLIGAFVILLLVV  207
             C  +   Q T  GC A+                  KFI+  N +A  L   F+   L +
Sbjct  162  SCRDSSGLQYT-QGCSAAL----------------DKFIE-KNILAVALC-VFLFAPLQI  202

Query  208  LFTILTVCI-------DVV  219
            L  +  VC+       +
Sbjct  203  LALVFAVCVCKAIQRGEDA  221
```
